# Structural Characterization and Expression Analysis of the *SERK/SERL* Gene Family in Rice (*Oryza sativa*)

**DOI:** 10.1155/2009/539402

**Published:** 2009-09-13

**Authors:** Bhumica Singla, Jitendra P. Khurana, Paramjit Khurana

**Affiliations:** Department of Plant Molecular Biology, University of Delhi South Campus, Benito Juarez Road, New Delhi 110021, India

## Abstract

Somatic embryogenesis (SE) is the developmental restructuring of somatic cells towards the embryogenic pathway and forms the basis of cellular totipotency in angiosperms. With the availability of full-length cDNA sequences from Knowledge-based Oryza Molecular Biological Encylopedia (KOME), we identified the leucine-rich repeat receptor-like kinase (LRR-RLK) genes from rice (*Oryza sativa*), which also encompasses genes involved in regulating somatic embryogenesis. Eight out of eleven of the rice *SERK* and *SERL* (*SERK-like*) genes have the TIGR annotation as (putative) brassinosteroid insensitive 1-associated receptor kinase (precursor). Real-time polymerase chain reaction analysis was undertaken to quantify transcript levels of these 11 genes. Most of these genes were upregulated by brassinosteroids although only a few of these displayed auxin induction. The expression profile of these genes is nearly uniform in the zygotic embryogenic tissue, but the expression pattern is more complex in the somatic embryogenic tissue. It is likely that *OsSERKs* and *OsSERLs* may be involved in somatic embryogenesis and also perform a role in morphogenesis and various other plant developmental processes. Functional validation of these somatic embryogenesis receptor-like kinase genes may help in elucidating their precise functions in regulating various facets of plant development.

## 1. Introduction

The ability to perceive and process information from chemical signals via cell surface receptors is a basic property of all living systems. The putative role of receptor-like kinases (RLKs), which belong to a large gene family [1], in developmental processes is to transduce environmental signals and/or information from the neighboring cells to trigger specific responses. One such developmental process is somatic embryogenesis which depends not only on the cell type employed but also on the culture conditions including composition of the nutrient medium [2].

SERK is a small gene family with at least five members in *Arabidopsis*, the model dicot plant [3]. Similarly, rice is considered a model plant among monocots [4]. Since the grasses are inferred to have monophyletic origin [5], information obtained from rice is invariably helpful for studying other cereal crops as well. Although earlier workers reported the isolation and characterization of *OsSERK* cDNA representing the first *SERK* from rice [6–8], the present study identifies and comprehensively analyzes the entire *SERK*-like (*SERL*) gene family from rice (*Oryza sativa*). This work involved the identification of *SERL* gene family members from rice, analysis of their structure, duplication, chromosomal distribution, and phylogenetic relationship. The real-time polymerase chain reaction (RT-PCR) analysis demonstrated that *SERK *and* SERL* genes are expressed differentially in developing seed, various organs/tissues, under light or dark, and are regulated by auxin/brassinosteroid treatment in rice, indicating that they may perform specific as well as redundant functions in plant development.

## 2. Material and Methods

### 2.1. Identification of OsSERK Gene Family in Rice

To identify *SERK* homologs in rice, the Knowledge-Based Oryza Molecular Biological Encylopedia (KOME) (http://cdna01.dna.affrc.go.jp/cDNA/), the National Centre for Biotechnology Information (NCBI) (http://www.ncbi.nim.nih.gov/BLAST/nr/EST), and The Institute for Genomic Research (TIGR) database (http://www.tigr.org/tdb/edb2/osa2/osa1/htmls/osa1.html) resources were used. Using the amino acid sequences of the available SERK proteins in TIGR and GenBank, polypeptide records of Viridiplantae, including all land plants and algae with a cut off E-value of 1 × 10^−10^, 55 sequences (NCBI, 25; TIGR, 30) were retrieved, downloaded, and used to search for their homologs in rice KOME using the TBLASTN program [9] after multiple alignment by ClustalX (version 1.83) program [10]. Additional members of rice *SERK* gene family were also searched by TBLASTN and BLASTN in the NCBI (nr and est) and TIGR databases, and 11 full-length cDNA sequences were recovered after removing the redundant sequences.

### 2.2. Sequence Analysis

The pseudomolecules of rice chromosomes available at TIGR (release 4) were used to position *OsSERK* and *OsSERL* genes by the BLASTN search. The number and position of exons and introns for individual *Oryza sativa* somatic embryogenesis receptor kinase (*OsSERK*) genes were determined by comparison of the cDNAs with their corresponding genomic DNA sequences. The multiple sequence alignment was carried out using ClustalX (version 1.83), and the unrooted phylogenetic tree was generated using neighbour-joining method. Bootstrap values from 1000 replicates are indicated at each node. The Gene Runner program (version 3.04), and DNASTAR (version 4.0) were used for the DNA and protein analysis.

### 2.3. Plant Material and Growth Conditions

Rice seeds (*O. sativa* L. ssp. *indica* var. Pusa Basmati 1) were soaked overnight in RO water after sterilization with 0.1% HgCl_2_ for 30 minutes. The tissues were harvested from 3/4-day-old seedlings grown on RO-soaked cotton, either in dark or in 14-hour light and 10-hour dark cycle in a culture room maintained at 28 ± 1°C. Floral tissue was collected from rice plants grown under field conditions. The callus tissue was raised as described [11]. For auxin treatment, the coleoptiles from 4-day-old etiolated rice seedlings were incubated in KPSC buffer (10 mM potassium phosphate, pH 6.0, 2% sucrose, 50 mM chloramphenicol) for 16 hours to deplete endogenous auxin; the buffer was changed every hour. The coleoptiles were then transferred to a fresh buffer with 30 *μ*M concentration of 2,4-dichlorophenoxy acetic acid (2,4-D) and incubated for 3 hours or with 100 nM epibrassinolide (EBR) for 1 hour [12].

### 2.4. RNA Isolation and Real-Time PCR

Total RNA was extracted using the RNeasy Plant mini kit (Qiagen, Germany) according to the manufacturer's instructions. The quantitative real-time PCR analysis was performed as described [12]. The primer sequences are listed in Supplementary Table 1(see Table 1 in Supplementary Material available online at doi: 10.1155/2009/539402). The specificity of the reactions was verified by melting curve analysis. The relative mRNA levels for each of the 11 *OsSERK* and *OsSERL* genes in RNA isolated from various tissue samples were quantified with respect to the internal standard, actin. At least two independent RNA isolations were used for cDNA synthesis, and each cDNA sample was subjected to real-time PCR analysis in triplicate.

## 3. Results and Discussion

### 3.1. SERK and SERK-Like (SERL) Gene Family in Rice

The *SERK* genes are present as small multigene family in *Arabidopsis* [3], maize [2], sunflower [13],* Poa pratensis* [14], rice [7], and wheat [15]. Nine nonredundant clones having high sequence similarity with known SERK proteins could be identified by TBLASTN or BLASTN search (International rice genome sequencing project; http://www.tigr.org/tdb/e2kl/osal/; http://www.rgp.dna.affrc.go.jp/). Thus, overall the *SERK *gene family in rice comprises of two members, *OsSERK1* and *OsSERK2* [6, 7], and the *SERK*-like (*SERL*) gene family comprises of nine members, designated as *OsSERL 1* to *9* (Table 1). Among the 11 members, most of them have been annotated as BRI-associated receptor-like kinase, precursor, putative BRI-associated receptor-like kinase and LRR family protein in TIGR.

### 3.2. Sequence Analysis of OsSERK and OsSERK-Like (OsSERL) Proteins

**Figure 1:**
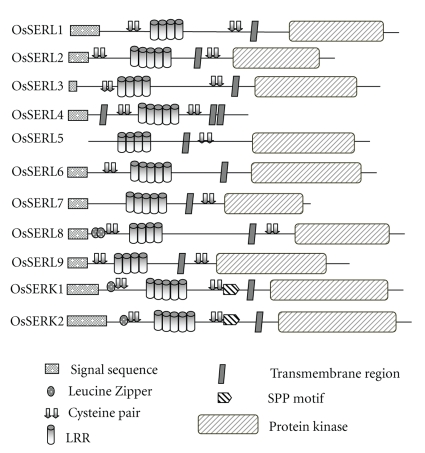
The domains are represented pictorially for the 2 OsSERK and 9 OsSERL proteins. To summarize, a signal peptide sequence is present in all except OsSERL5, one cysteine pair is present in OsSERL5, OsSERL6, and OsSERL7, five LRRs are present in OsSERK1, OsSERK2, OsSERL2, OsSERL4, OsSERL6, and OsSERL7, trans-membrane domain is present in all, SPP motif is present in only two, OsSERK1 and OsSERK2, and a protein kinase domain is present in all except OsSERL4.

The deduced molecular mass of rice SERK and SERL peptides ranges from 60 kDa for OsSERL5 to 73 kDa for OsSERL8, except OsSERL4 which lacks the kinase domain (Table 1). To examine in detail the domain organization of OsSERL proteins, the multiple sequence alignments of the full-length protein sequences were done using the ClustalX program (see Supplementary Figure 1). Among the 11 proteins described, two showed the presence of the SPP motif, and the remaining nine showed the characteristic features of the SERK proteins, except the SPP motif. On the basis of domain search programmes (http://us.expasy.org/prosite/; http://www.ebi.ac.uk/InterProscan/; http://smart.embl/heidelberg.de/; http://www.sanger.ac.uk/software/pfam/index.html), the deduced domains are represented in a pictorial form (Figure 1). To summarize, a signal peptide sequence is present in all except OsSERL5, one cysteine pair is present in OsSERL5, OsSERL6, and OsSERL7, five LRRs are present in OsSERK1, OsSERK2, OsSERL2, OsSERL4, OsSERL6, and OsSERL7, transmembrane domain is present in all, SPP motif is present in only two, OsSERK1 and OsSERK2, and a protein kinase domain is present in all except OsSERL4. Since OsSERL4 and OsSERL7 were showing significant homology (75%) at the protein level but OsSERL4 was lacking the kinase domain and also harbouring an unusually long 3′UTR (1552 bp), the sequence of the 3′UTR was aligned with the cDNA region coding for the kinase and C-terminal domain of OsSERL7. It could be clearly deduced from the alignment that OsSERL4 has lost the kinase activity due to changes in certain nucleotide positions and also some changes in the respective frame; even in the absence of the kinase domain the 3′UTR depicts relic of the original kinase domain. Receptor protein kinases are plasma membrane-bound and play an important role in the perception and transmission of external signals [16, 17]. The protein localization of these genes as shown by the PSORT programme suggests that 9 out of 11 proteins reside in the plasma membrane (OsSERL1, OsSERL2, OsSERL3, OsSERL4, OsSERL6, OsSERL7, OsSERL8, OsSERK1 and OsSERK2), which is the case with most of the SERKs also [18]. On the other hand, OsSERL5 and OsSERL9 are localized to the inner mitochondrial membrane and endoplasmic reticulum, respectively.

### 3.3. Gene Structure and Phylogenetic Analysis of Rice SERK and SERL Genes

**Figure 2:**
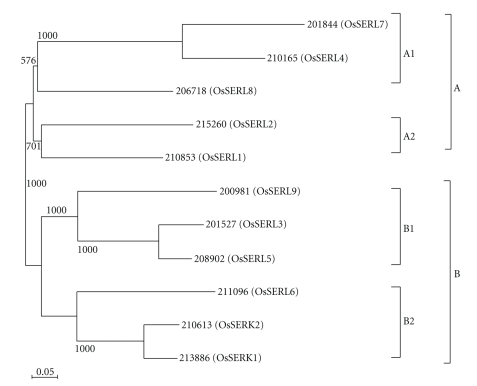
Phylogenetic relationship among the rice SERK and SERL proteins. The unrooted tree was generated using ClustalX program by neighbour-joining method. Bootstrap values from 1000 replicates are indicated at each node.

A pairwise analysis of the full-length OsSERK protein sequences indicated that the overall identities among the OsSERK and OsSERL proteins range from 35% to 87% and from 25% to 87% with the *Arabidopsis* SERK proteins, respectively. On comparing the full-length cDNA sequences with the corresponding genomic DNA sequence, it appears that the coding sequences of the majority of the *OsSERL *genes (7 among 9) have 10-11 introns (Table 1). To examine the phylogenetic relationship among the rice SERK and SERL proteins, an unrooted tree was constructed from alignments of the full-length SERK protein sequences (Figure 2). The sequences formed separate clusters and were grouped into two major groups (groups A and B) with well-supported bootstrap values. Five and six OsSERK and OsSERL proteins were included in groups A and B, respectively. Groups A and B could be further sub-divided into two subgroups each (A1-A2 and B1-B2) with varying degree of bootstrap support. Most of the genes grouped together showed conserved gene structure, in terms of exon/intron organization and intron phasing. Eleven of the proteins formed four sister-pairs, two of them showing very strong bootstrap support (100%). The phylogenetic tree with the SERK family in *Arabidopsis*, maize, sunflower, *Poa pratensis*, *Gossypium hirsutum*, and rice clustered the SERLs and the rest of the SERKs separately (see Supplementary Figure 2).

### 3.4. Differential Expression of OsSERK and OsSERL Genes

**Figure 3:**
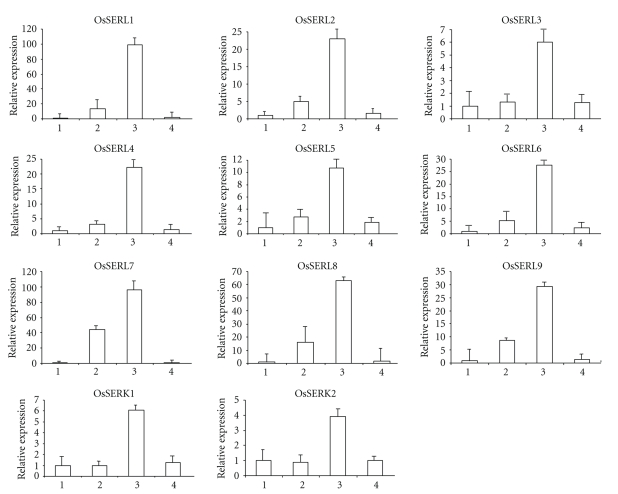
Real-time PCR expression profiles of individual *SERK* and* SERL* genes. (1) The relative mRNA levels of individual *OsSERK* and *OsSERL *genes in pre-pollinated, (2) 1-2 days after pollination, (3) 5-10 days after pollination, and (4) 21 days after pollination from field grown rice seedlings. The relative mRNA levels of individual *OsSERK* and *OsSERL* genes normalized with respect to housekeeping gene *Actin. *

**Figure 4:**
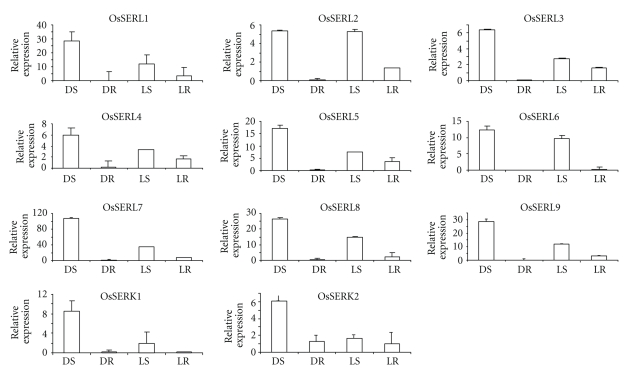
Real-time PCR expression profiles of individual *SERK* and* SERL* genes. The relative mRNA levels of individual *OsSERK* and *OsSERL* genes are normalized with respect to housekeeping gene *Actin* in different tissues (*DS* dark shoot, *DR* dark root, *LS* light shoot, and* LR* light root).

To determine the organ-specific expression of each *OsSERK* and *OsSERL* genes, real-time PCR was performed with total RNA isolated from different stages of rice seed development (days after pollination; DAP) (Figure 3) as well as dark-grown roots, etiolated shoots, green shoots, and roots (Figure 4). This analysis revealed that in all 11 cDNAs of rice, expression is maximum at 5–10 DAP (Figure 3). In case of OsSERL proteins, there was a gradual increase in the expression level starting from prepollination to 5–10 DAP. At this stage, the embryonic phase and juvenile phase coexist, and enlargement and formation of embryonic organ continues [19]. *OsSERL1* and *OsSERL7* were the highest expressing followed by *OsSERL8*. *OsSERK1*, *OsSERK2,* and *OsSERL3* depicted low activity as compared to the *OsSERLs*. At 21 DAP (developing seed), the expression decreased in all the genes examined (Figure 3). It has been reported that in *Arabidopsis*, *AtSERK1* expressed in flowers at 3 DAP, containing developing seeds with embryos from stages 1 through 7 [20], although after fertilization *AtSERK1* expression appeared to decrease rapidly and was detected in few cells of the developing seed [3]. Essentially similar has been the case for *CiSERK1* transcripts which are highly abundant in fruits at 30 DAF and 60 DAF but not at 180 DAF [21]. *PpSERK1* expression was high during premeiosis and decreased during meiosis and postmeiosis [14]. It appears that, in general, SERKs and SERLs expression gradually decreases at the later stages, after fertilization. *OsSERK1* and *OsSERK2* show the least expression in various stages of developing seed as compared to *OsSERLs*. Thus, it appears that the expression of *OsSERK* genes is probably confined or is more specific in somatic embryogenesis than in zygotic embryogenesis where they play a constitutive role as is evident from our data. Studies would be, however, required to check the expression pattern prior to fertilization in ovules as *AtSERK1*, *ZmSERK1*, *ZmSERK2*, and *PpSERK2* show high expression in developing ovules [2, 3, 14]. 

**Figure 5:**
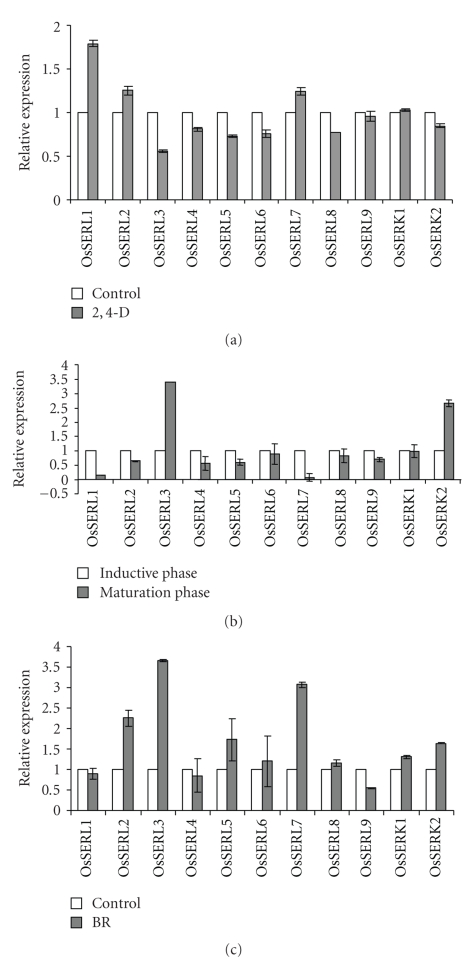
The relative mRNA levels of the *SERK/SERL* genes in (a) 3-day-old etiolated seedlings in either water or 30*μ*M 2,4-D, (b) embryogenic calli during induction and maturation phases, and (c) 3-day-old etiolated rice coleoptiles treated with 100 nM BR.

 To check the expression in different organs of rice, total RNA from roots (dark and light grown), etiolated and green shoots, was isolated. *OsSERK* and *OsSERL* genes show a complexity of specific and overlapping expression patterns in various organs/tissues analyzed. Expression of *OsSERKs* and *OsSERLs* is higher in etiolated shoots (DS) than in green shoots (LS), except *OsSERL2*, which shows the same expression level in the two types of shoots, and all the *OsSERLs* show higher expression in light-grown roots (LR) than in dark-grown roots (DR); expression of the two *OsSERKs* is similar in light- and dark-grown roots: very low for *OsSERK1* and higher for *OsSERK2* (Figure 4). Exogenous auxin plays an important role in somatic embryo induction [22, 23]. For *OsSERL* genes, the effect of auxin was not significant, except a distinct upregulation for *OsSERL1*,* OsSERL2*, and *OsSERL7* (Figure 5(a)). Earlier studies have shown that auxin up-regulates *MtSERK1* expression in both *Medicago trunculata *root forming and embryogenic cultures [24]. The *OsSERK* and *OsSERL* genes showed complexity of specific and overlapping expression patterns in tissues or calli treated with the hormone auxin indicating that they might perform specific functions or act redundantly. Since SE can be categorized into inductive and maturation phase, it was imperative to investigate the SERK expression subsequent to auxin application. The difference in kinetics between individual *OsSERK* and* OsSERL* genes is likely due to a variety of factors, such as tissue-specific auxin perception, cell-type dependence and differential regulation of auxin concentration, or different modes of action of auxin-dependent transcriptional and posttrancriptional regulation. Among the two *SERKs*, *OsSERK2* showed specific expression during the maturation phase (Figure 5(b)), and it appears to be more specific for somatic embryogenesis than *OsSERK1*. Earlier reports also support this observation [6]. With the exception of *OsSERL3*, all *OsSERLs* were downregulated, with *OsSERL1* and *OsSERL7* being most affected during the maturation phase (having a broader role in morphogenesis), and *OsSERK2 *and *OsSERL3* were upregulated (probably being specific to SE). The *OsSERLs* lacking the characteristic SPP motif, except *OsSERL3*, showed a decrease in transcript in the embryogenic calli (maturation phase), thus further supporting the fact that the presence of the SPP motif may be the essential factor for an SERK function. *OsSERLs* seem to have a broader role in morphogenesis in cultured tissue rather than being specific to somatic embryogenesis. Essentially similar observations have been made for *TcSERK* in cacao [25] and for *MtSERK* in *Medicago trunculata* [24]. In sunflower too, *SERK* transcripts accumulate early after the beginning of the culture in the morphogenetic zone of immature zygotic embryos (IZEs) of sunflower, whatever the induction conditions used, that is, organogenic, embryogenic, or highly embryogenic conditions [13]. 

In plants, systemic signaling molecules and hormones play key roles in establishing the developmental program and are also intimately involved in shaping plant growth and development. Studies have demonstrated a link between brassinosteroids and auxin indicating that some pathways are under dual control [26–28], and some signaling pathways converge at the level of the transcriptional regulation of common target genes [28, 29]. It has been proposed that BR-induced effects are mediated via auxin or enhancing sensitivity to auxin [26, 30, 31] and several researchers have provided insights into auxin and BR interactions [32, 33]. Thus, to check the inducibility of these *OsSERK* and *OsSERL* genes by exogenous BR, their expression was examined by real-time PCR, and eight of these genes were found to be induced in coleoptiles treated with BR (Figure 5(c)); maximum induction was recorded in *OsSERL3* and *OsSERL7*. Also, in the last few years, studies have revealed the interaction between SERKs and other proteins like brassinosteroid insensitive (BRI) [34, 35]. Besides BRI receptor and its coreceptor, the AtSERK3 or BRI-associated kinase 1 protein, the *Arabidopsis* somatic embryogenesis receptor-like kinase protein complex has been shown to include kinase associated protein phosphatase, CDC48A, 14-3-3*ν*, MADS box transcription factor AGAMOUS-LIKE15, and an uncharacterized zinc finger protein, a member of the CONSTANS family [35]. Further studies would be, however, required to unravel the role of this protein complex and the interactive effect of brassinosteroids and auxins in regulating somatic embryogenesis.

## 4. Conclusions

*OsSERLs* seem to have a broader role in morphogenesis rather than being specific to somatic embryogenesis. Functional validation of these somatic embryogenesis receptor-like kinase genes may help in elucidating their precise functions in regulating various facets of plant development.

## Supplementary Material

Supplementary Figure 1. Multiple alignments of the full-length rice SERK and SERL proteins obtained with ClustalW. Fully and partially conserved (present in more than 50% of aligned sequences) residues are highlighted in black and grey boxes, respectively. Gaps (marked with dashes) have been introduced to maximize the alignments.
Supplementary Figure 2. Phylogenetic relationship among the rice SERK, SERL, *Arabidopsis* (AtSERK1 to AtSERK5; CAB42254, AAK68073, AAK68074, AAD28318 and AAD28319), maize (ZmSERK1, ZmSERK2 and ZmSERK3; CAC37638, CAC37639 and CAC37642), sunflower (HaSERK1 to HaSERK4; AAL93161, AAL93162, AAL93163 and AAL93164), *Poa pratensis* (PpSERK1 and PpSERK2; AJ841693 and AJ841697) and *Gossypium hirsutum* (GhRLK1 and GhRLK2; AAT64017 and AAT64032) proteins.
Supplementary Table 1. Sequences of Real-Time PCR primers.

## Figures and Tables

**Table 1:** *OsSERK* and *OsSERL* gene family in rice.

Name	Clone ID^(a)^	Accession number^(b)^	Locus Id^(c)^	CDS length (bp)^(d)^	Protein length (aa)^(e)^	ORF length (bp)^(f)^	5′ Prime UTR (bP)^(g)^	3′ Prime UTR (bp)^(h)^	Mol wt (Da)^(i)^	pI^(j)^	No. of introns^(k)^	Genomic Locus^(l)^				Nearest markr^(m)^
												BAC/PAC	Chromosome	Accession	cM	
												name	number	number	position	

OsSERL1	200981	AK111536	Loc_Os11g39370	2511	608	1827	215	470	67733.58	6.99	11	OSJNBb0030E22	11	AC120884	91.4	G4001
OsSERL 2	201844	AK066417	Loc_Os06g16330	2500	644	1935	169	397	69308.82	6.65	9	P0676F10	6	AP005813	54.1	S2539, C235, P138
OsSERL 3	210165	AK073972	Loc_Os02g49600	2639	324	975	113	1552	35295.7	8.07	8	OSJNBa0072H09	2	AP00575	129.4	C12691, C10187S
OsSERL 4	215260	AK120541	Loc_Os02g14120	2313	620	1863	160	291	67789.62	6.6	10	OJ1077_A12	2	AP003991	36.8–37.0	C2168, S10927S
OsSERL 5	210853	AK100017	Loc_Os1g07630	2421	628	1887	259	276	68913.90	6.59	10	P0583G08	1	AP003282	16.4	E222S
OsSERL 6	211096	AK100258	Loc_Os06g12120	2438	616	1851	425	163	67721.09	5.97	10	P0638H11/	6	AP003513	34.3	Y2587L
OsSERL 7	206718	AK111771	Loc_Os07g12320	2671	678	2037	126	509	72952.82	7.13	10	P0708B04	8	AP004764	80.7–82.8	S11114, E60162SB
OsSERL 8	208902	AK111846	Loc_Os03g49620	2507	543	1632	699	137	59976.31	5.73	11	OSJNBa0004L11	3	AC133334	122.8	R2847
OsSERL 9	201527	AK066118	Loc_Os02g18320	2968	607	1824	752	393	67658.71	5.44	10	OSJNBa0018M09	2	AP005533	50.3	C12706S, C626
OsSERK 1	213886	AK103038	Loc_Os08g07760	2674	624	1875	286	514	68701.34	6.21	10	OSJNBa0054L03	8	AP005164	34.6–35.7	E60560S, R2976
OsSERK 2	210613	AK099777	Loc_Os04g38480	2296	628	1887	203	207	68587.33	6.31	10	OSJNBa0036B21	4	AL606636	68.3	E3080S

^(a)^Systematic designation given to rice SERK clone. ^(b)^Accession number of full-length cDNA sequence from KOME (http://cdna01.dna.affrc.go.jp/cDNA/). ^(c)^Locus ID of each *OsSERK *and *OsSERL* gene on rice chromosome psuedomolecules available at TIGR (release 4). ^(d)^Length of complete cDNA sequence in base pairs. ^(e)^Length (number of amino acids (aa)) of the deduced polypeptide. ^(f)^Length of open-reading frame in base pairs. ^(g,h)^Length of 5 prime and 3 prime UTR in base pairs. ^(i,j)^Molecular weight (Daltons) and isoelectric point (pI) of the deduced polypeptides. ^(k)^Number of introns present within ORF. ^(l)^Name, chromosome number, accession number, and approximate cM position of the BAC/PAC clone in which SERK gene is present. ^(m)^Nearest marker to the SERK gene.
