# Supplementary material for: Structural Characterization and Expression Analysis of the SERK/SERL Gene Family in Rice (Oryza sativa)
Source: Int J Plant Genomics. 2009 Sep 13;2009:539402. doi: 10.1155/2009/539402 (PMC2742738; doi:10.1155/2009/539402)
Supplement: Supplementary file 1 — Supplementary Figure 1. Multiple alignments of the full-length rice SERK and SERL proteins obtained with ClustalW. Fully and partially conserved (present in more than 50% of aligned sequences) residues are highlighted in black and grey boxes, respectively. Gaps (marked with dashes) have been introduced to maximize the alignments. Supplementary Figure 2. Phylogenetic relationship among the rice SERK, SERL, Arabidopsis (AtSERK1 to AtSERK5; CAB42254, AAK68073, AAK68074, AAD28318 and AAD28319), maize (ZmSERK1, ZmSERK2 and ZmSERK3; CAC37638, CAC37639 and CAC37642), sunflower (HaSERK1 to HaSERK4; AAL93161, AAL93162, AAL93163 and AAL93164), Poa pratensis (PpSERK1 and PpSERK2; AJ841693 and AJ841697) and Gossypium hirsutum (GhRLK1 and GhRLK2; AAT64017 and AAT64032) proteins. Supplementary Table 1. Sequences of Real-Time PCR primers. [file 539402.f1.pdf]

Supplementary Figure 1

```
                              Signal Sequence                          Leucine zipper
213886    ------MAAHRWAVWAVLLLRLLVPAAR-------VLANMEGDALHSLRTNLVDPNNVLQ 47
210613    MAEARLLRRRRLCLAVAVVWVVAVAVSR-------VGANTEGDALYSLRQSLKDANNVLQ 53
211096    ------MAARRRLLLLLVLLLCRLAA---------VLPTSEVEALQGFMAGFAGGNAAFQ 45
208902    ------------------------MPSC-------R------------------------ 5
201527    ----------MRELRVAVLIIAVSLPSF-------SASDRQGDALYDMKQKLNVTGNQLS 43
200981    ----------MKLIAFGLLLLG-YIQYF-------ATPDSEVIALYEIRTMLNDSRGVLN 42
210853    ------MRMRWWAAPLAAVLAVILLPSS-TATLSPAGINYEVVALMAIKTELQDPYNVLD 53
215260    -----MAGPRALAVAAAVVVAAWAVAAAGDPPLSPKGLNYEVAALMAVKSRMRDEKGVMG 55
206718    -----MASNLFLLLFFLVVSYAPFLAFS------SEPLNPEVEALIAIRQGLVDPHGVLN 49
210165    ----MAMEVALAVYSLVLLASFSFPCRLASALLSPKGVNYEVQALMMIKTSLKDPHGVLK 56
201844    ------MEPPFFLLLLLLVVSSSSPS---AALLSAKGVYNEVQALIVIKNLLKDPHGVLK 51
                                                    .  :

                                                LRR1                    LRR2
213886    SWDPTLVNPCTWFHVTCNNDNSVIRVDLGNAALSGTLVPQLGQLKNLQYLELYSNNISGT 107
210613    SWDPTLVNPCTWFHVTCNPDNSVIRVDLGNAQLSGALVPQLGQLKNLQYLELYSNNISGT 113
211096    SWDASAPNPCTWFHVTCGPGNQVIRLDLGNQSLSGELKPDIWQLQALQSLELYGNSISGK 105
208902    --------------------------TLASMGFTGVLSPRIGELQFLNVLTLPGNKITGG 39
201527    DWNQNQVNPCTWNSVICDNNNNVIQVTLAARGFAGVLSPRIGELKYLTVLSLAGNRISGG 103
200981    GWNNNQVSPCYFPSISCNQDQKVISITLSSSGLSGFLSPSIGKLLYLQQLLLNDNNITGG 102
210853    NWDINSVDPCSWRMVTCSADGYVSALGLPSQSLSGKLSPGIGNLTRLQSVLLQNNAISGT 113
215260    GWDINSVDPCTWSMVACSPDGFVVSLQMANNGLAGTLSPSIGNLSHLQTMLLQNNMISGG 115
206718    NWDEDSVDPCSWAMVTCSAHNLVIGLGAPSQGLSGTLSGRIANLTNLEQVLLQNNNITGR 109
210165    NWDQDSVDPCSWTMVTCSPENLVTGLEAPSQNLSGLLSASIGNLTNLEIVLLQNNNINGP 116
201844    SWDQNSVDPCSWAMITCSPDFLVTGLEAPSQHLSGLLSPSIGNLTNLETVLLQNNNITGP 111
          . .    ...     ..                ::* *    : :*   *   : *  .* *.*

                                   LRR3                      LRR4
213886    IPSELGNLTNLVSLDLYLNNFTGPIPDSLGNLLKLRFLRLNNNSLSGSIPKSLTAITALQ 167
210613    IPNELGNLTNLVSLDLYLNNFTGFIPETLGQLYKLRFLRLNNNSLSGSIPKSLTNITTLQ 173
211096    IPSELGRLASLQTLDLYLNNFTGEIPNELGNLSKLSNLRLNNNSLSGAIPMSLTTIQNLE 165
208902    IPEQIGNLSSLTSLDLEDNLLVGPIPASLGQLSKLQILILSQNNLNGTIPDTVARISSLT 99
201527    IPEQFGNLSSLTSLDLEDNLLVGEIPASLGQLSKLQLLILSDNNFNGSIPDSLAKISSLT 163
200981    IPQELGNLSSLTTLKLGGNSLNGSIPDSLGRLSKLQNLDMSKNLLIGNIPTSLSNLSSLN 162
210853    IPASIGRLGMLQTLDMSDNQITGSIPSSIGDLKNLNYLKLNNNSLSGVLPDSLAAINGLA 173
215260    IPPEIGKLTNLKALDLSGNQFVGEIPSSLGRLTELNYLRLDKNNLSGQIPEDVAKLPGLT 175
206718    LPPELGALPRLQTLDLSNNRFSGRVPDTLGRLSTLRYLRLNNNSLSGAFPSSLAKIPQLS 169
210165    IPEEIGRLTKLKTLDLSSNHFSGGIPNSVGHLESLQYLRLNNNTLSGAYPSSSANLSQLV 176
201844    IPAEIGRLENLKTLDLSSNSFYGEIPSSVGHLESLQYLRLNNNTLSGPFPSASANLSHLV 171
          :*  .:*  *   *  :*.:    *  :  *  :*   :*  *   *   *  :..*  :  *   *    :  :   *

                     LRR5                                           SPP Motif
213886    VLDLSNNNLSGEVPSTGSFSLFTPISFANNPSLCGPGTTKPCPGAPPFSPPPPYNPPTP- 226
210613    VLDLSNNNLSGEVPSTGSFSLFTPISFANNKDLCGPGTTKPCPGAPPFSPPPPFNPPTP- 232
211096    VLDLSHNNLSGIIPTNGSFSHFTPISFSNNPRTFANSSDSPSNNSGAAVPS--------- 216
208902    DIRLAYNKLSGSIP--GSLFQVARYNFSGNNLTCGANFLHPCSSSISYQGS--------- 148
201527    DIRLAYNNLSGQIP--GPLFQVARYNFSGNHLNCGTNFPHSCSTNMSYQSG--------- 212
200981    DINLADNNLSGEIP--KRLLQVSHYSYIGNHLNCGQHLIS-CEGNNINTGG--------- 210
210853    LVDLSFNNLSGPLP----KISSRTFNIVGNPMICGVKSGDNCS--SVSMDPLS------- 220
215260    FLDLSSNNLSGPVP----KIYAHDYSIAGNRFLCNSSIMHGCKDLTVLTNEST------- 224
206718    FLDLSYNNLTGPVP----HFPTRTFNVVGNPMICGSSSGSHAGNANAAECATVVAPVTVP 225
210165    FLDLSYNNLSGPVP----GSLARTFNIVGNPLICAAGTEHDCYGTLPMPMSYSLNNTQGT 232
201844    FLDLSYNNLSGPIP----ESLARTYNIVGNPLICDANREQDCYGTAPMPMSYSLNGSRGG 227
           :  *:  *:*:*  :*:.          .   .*           .
```

```
                               Transmembrane Domain

213886    ---VQSPGSSSSTGAIAG----GVAAGAALLFAI-----------PAIGFAWYRRR-KPQ 267
210613    ---TVSQGDSK-TGAIAG----GVAAAAALLFAV-----------PAIGFAWWRRR-KPE 272
211096    -----GRSSASSIGTIAG----GAAAGAAMLFAA-----------PIVLFAWWWRR-KPH 255
208902    -------SHGSKVGIVLG----TVVGAIGILIIG-----------AVFIVCNGRRKSHLR 186
201527    -------SHSSKIGIVLG----TVGGVIGLLIVA-----------ALFLFCKGRRKSHLW 250
200981    -------SNNSKLKVVA-----SIGGALTLLVII-----------VLFLLWWQRMR-HRP 246
210853    ------YPPDDLKTQPQQ----GIARSHRIAIICGVTVGSVAFATIIVSMLLWWRHRRNQ 270
215260    ------ISSPSKKTNSHH----QLALAISLSIIC---------ATVFVLFVICWLKYCRW 265
206718    FPLDSTPSSSSRAAAAAVGRSKGGGGAARLPIGVGTSLGASALVLLAVSCFLWRRRRRHR 285
210165    LMP--AKSKSHKVAIAFG----STIGCISFLIPVMG-------------LLFWWRHRRNH 273
201844    ALPPAARDRGHKFAVAFG----STAGCMGLLLLAAG-------------FLFWWRHRRNR 270
                                         : .                       :

                                                                 I

213886    EHF---------------FDVPAEEDPEVHLGQLKRFSLRELQVATDTFSNKNILGRGGF 312
210613    EHF---------------FDVPAEEDPEVHLGQLKRFSLRELQVATDNFSNKNILGRGGF 317
211096    DQF---------------FDLLEEETPEVHLGQLRRFTLRELQVATDNFSQTNLLGRGGF 300
208902    EVF---------------VDVSGEDDRRIAFGQLKRFAWRELQLATDSFSEKNVLGQGGF 231
201527    EVF---------------VDVAGEDDRRIAFGQLKRFAWRELQIATDNFSERNVLGQGGF 295
200981    EIY---------------VDVPGQHDHNLEFGQIKRFSLRELQIATNNFSEQNVLGKGGF 291
210853    QIF---------------FDVNDQYDPEVCLGHLKRYAFKELRAATNNFNSKNILGEGGY 315
215260    RLP---------------FASADQ-DLEIELGHLKHFSFHELQSATDNFNSKNILGQGGF 309
206718    CLLSGPSSVLGILEKGRDVEDGGGGEVMARLGNVRQFGLRELHAATDGFSARNILGKGGF 345
210165    QIL---------------FDVD----------------------------GK-------- 282
201844    QIL---------------FDVDEQQIENVNLGNVKRFSFRELQAATEGFSGKNILGKGGF 315
                            .            .        .   ::.  .  .  . ..

                            II          III              IV          V

213886    GKVYKGRL--ADGSLVAVKRLKEERTPGGELQFQTEVEMISMAVHRNLLRLRGFCMTPT- 369
210613    GKVYKGRL--ADGSLVAVKRLKEERTPGGELQFQTEVEMISMAVHRNLLRLRGFCMTPT- 374
211096    GKVYKGRL--LDGSLIAIKRLNEDRIGTGERQFLMEVEIISMAVHQNLLRLQGYCMTPT- 357
208902    GKVYKGAL--PDGTKIAVKRLTDYESPGGEAAFLREVELISVAVHRNLLRLIGFCTTQT- 288
201527    GKVYKGVL--PDGTKIAVKRLTDYESPGGEAAFLREVELISVAVHRNLLKLIGFCTTQT- 352
200981    GKVYKGVLSGPHGRKVAVKRLFEVEKPEGEIAFLREVELISIAVHKNILRLIGFCTTTK- 350
210853    GIVYKGFLR--DGAIVAVKRLKDYNAVGGEVQFQTEVEVISLAVHRNLLRLIGFCTTEN- 372
215260    GVVYKGCLR--NGALVAVKRLKDPDITG-EVQFQTEVELIGLAVHRNLLRLYGFCMTSK- 365
206718    GDVYRGRLS--DGTVVAVKRLKDP-TASGEAQFRTEVEMISLAVHRHLLRLVGFCAAASG 402
210165    ----------QKAKLLSIMPQCSSNLG---AYWH---AMVVFIVTHWLLQSN-------- 318
201844    GNVYRGQL--PDGTLMAVKRLKDGNAAGGEAQFQTEVEMISLALHRNLLRLYGLLHDRHR 373
          .    .     ..  :::    .     ..  :  .  ::  . : : :*:  .

                                                                 VI

213886    ERLLVYPYMANGSVASRLRERPPSEPPLDWRTRRRIALGSARGLSYLHDHCDPKIIHRDV 429
210613    ERLLVYPYMANGSVASRLRERQPNDPPLEWQTRTRIALGSARGLSYLHDHCDPKIIHRDV 434
211096    ERLLVYPYMENKSLETRLRECSDSQQPLDWPTRRKIALGSARGISYLHEGCDPKIIHRDV 417
208902    ERLLVYPFMQNLSVAYRLREFKPGEPILDWSARKRVAIGTARGLEYLHEHCNPKIIHRDV 348
201527    ERLLVYPFMQNLSVAYRLRDFKPGEPVLNWPERKRVAIGTARGLEYLHEHCNPKIIHRDV 412
200981    ERLLVYPYMENLSVASRLRDIKLNEPALDWPTRVRIALGAARGLEYLHEHCNPKIIHRDV 410
210853    ERLLVYPYMPNGSVASQLRELVNGKPALDWSRRKRIALGTARGLLYLHEQCDPKIIHRDV 432
215260    ERLLVYPYMPNGSVADRLRDYHHGKPSLDWSKRMRIAVGAARGLLYLHEQCNPKIIHRDV 425
206718    ERLLVYPYMPNGSVASRLR----GKPPLDWQTRKRIAVGTARGLLYLHEQCDPKIIHRDV 458
210165    --------TQRMST---------------------------------------------- 324
201844    APPSLPLHVQRQRRLPPQSEAGVGVGDEEEDRGGGGEGPGVPARAVRPQDHPPRRQGRQR 433
                    .        .   .    .             .     .   .     .
```

```
                                        VII                 VIII              IX
213886      KAANILLDEDFEAVVGDFGLAKLMDYKDTHVTTAVRGTIGHIAPEYLSTGKSSEKTDVFG 489
210613      KAANILLDEDFEAVVGDFGLAKLMDYKDTHVTTAVRGTIGHIAPEYLSTGKSSEKTDVFG 494
211096      KAANILLDEKLEAVVGDFGLARIMDYKVSHVVTGVMGTLGHIPMEYLTAGRTSDKTDVFG 477
208902      KAANVLLDEDFEPVVGDFGLAKLVDVQKTSVTTQVRGTMGHIAPEYLSTGKSSERTDVFG 408
201527      KAANVLLDEDFEPVVGDFGLAKLVDVQKTSVTTQVRGTMGHIAPEYLSTGKSSERTDVFG 472
200981      KAANVLLDGNFEAVVGDFGLAKMIDRERNTVTTGVRGTMGHIAPEYLKTGRPSVKTDIFG 470
210853      KASNVLLDEYFEAIVGDFGLAKLLDHRESHVTTAVRGTVGHIAPEYLSTGQSSEKTDVFG 492
215260      KAANILLDESFEAIVGDFGLAKLLDRQESHVTTAVRGTIGHIAPEYLSTGQSSEKTDVYG 485
206718      KAANVLLDECHEAVVGDFGLAKLLDHGDSHVTTAVRGTVGHIAPEYLSTGQSSEKTDVFG 518
210165      ------------------------------------------------------------
201844      AAGRGVRGRRRRLRAGQAAGPPGVARHHGGAGHRRPHRAGVPVHGAVVGPDRRLRLRHPA 493
             :.     .        .. .  .                   .               .

                                 X
213886      YGIMLLELITGQRAFDLAR----LANDDDVMLLDWVKGLLKEKRLEMLVDPDLQSNY--- 542
210613      YGIMLLELITGQRAFDLAR----LANDDDVMLLDWVKGLLKEKKVEMLVDPDLQSGF--- 547
211096      YGIMLFELISGKRGFDLVG----LANEENARVHDWVKKLLEEDRLEVLIDPNLLEIYNGG 533
208902      YGIMLLELVTGQRAIDFSR----LEEEDDVLLLDHVKKLQREGQLGAIVDRNLSSNYD-- 462
201527      YGIMLLELVTGQRAIDFSR----LEEEDDVLLLDHVKKLQREGQLGSIVDRNLNQNYD-- 526
200981      YGVMLLEIVTGERAVFPEF----SEGDSEIMLNDQVKRLVQGGRLTDIVDHNLDTAYD-- 524
210853      FGVLLVELITGQKALDFGR----LANQ-KGGVLDWVKKLHQEKQLSMMVDKDLGSNYD-- 545
215260      FGILLLELITGPKTLSNGH----AQSQ-KGMILDWVREVKEENKLDKLVDRDLKYSFD-- 538
206718      FGILLLELVTGQRALEVGKGS-GVIQHQKGVMLDWVRKVHQEKLHDLLVDQDLGPHYD-- 575
210165      ------------------------------------------------------------
201844      AGARHRPDGARVRQVVQPQGRHAGLGEEDAVGEEGGGAGGQGVRRRVRPGGGGGDGAGGS 553
              .         :                  ..      .              . .     ...

                              XI                      C- Terminal Domain
-
213886      ---IDVEVESLIQVALLCTQGSPTERPKMAEVVRMLEG-DGLAERWEEWQKIEVVRQEVE 598
210613      ---VEHEVESLIQVALLCTQGSPMDRPKMSEVVRMLEG-DGLAERWEEWQKVEVVRQEAE 603
211096      EQGVREEMRLLVQIALLCTQESAPSRPRMSTVVTMLE--DGIAEHWDAWQRKTIVQASLQ 591
208902      ----GQEVEMMIQIALLCTQASPEDRPSMSEVVRMLEG-EGLAERWEEWQQVEVTRRQDY 517
201527      ----DEEVEMMIQIALLCTQSSPEDRPSMSEAVRMLEG-EGLAERWEEWQQVEVTRRQEY 581
200981      ----LQQLEKMIQIALLCTHVEPHLRPAMSEVVQMLEGNVVPAEQWEEWQVAELARRHQH 580
210853      ----RVELEEMVQVALLCTQYYPSHRPRMSEVIRMLEG-DGLAEKWEASQNVDTPKSVSS 600
215260      ----FAELECSVDVILQCTQTNPILRPKMSEVLNALEA-NVTLPENGIDLNREVPPYGGS 593
206718      ----RIEVAEMVQVALLCTQFQPSHRPRMSEVVRMLEG-DGLAEKWEANHRPAAMAAAAA 630
210165      ------------------------------------------------------------
201844      PLHPVPPRPPPPHVRRRPHARGRRPRRPLGEGLRPLHRRRRLPLPLPPHLRPGAARRRLR 613
                                           .

213886      LGPHRNS--EWIVDSTDN--------------------LHAVELSGPR 624
210613      LAPRHN---DWIVDSTYN--------------------LRAMELSGPR 628
211096      GGQGVS---EARNDSVAN--------------------LPPDTLSGPR 616
208902      ERMQQR--FDWGEDSIFN--------------------QEAIELSAGR 543
201527      ERMQRR--FDWGEDSVYN--------------------QEAIELSGGR 607
200981      EMNQQRRLFSFSEESLNI--------------------QEAIQLSSGR 608
210853      ELLPPKFMDFAADESSLG--------------------LEAMELSGPR 628
215260      CSFSVRHED-PHDSSSFI--------------------IEPIELSGPR 620
206718      PHELGYDHRNDSNGSVFFNDFHDNDSSLSSDEVRSIDMVEEMELSGPR 678
210165      ------------------------------------------------
201844      RRLRPLLLRPHRRLLPPRPGRR----------------ALRPQITPP- 644
                                                         :
```
