# Supplementary figures and images for: Structural Characterization and Expression Analysis of the SERK/SERL Gene Family in Rice (Oryza sativa)
Source: Int J Plant Genomics. 2009 Sep 13;2009:539402. doi: 10.1155/2009/539402 (PMC2742738; doi:10.1155/2009/539402)

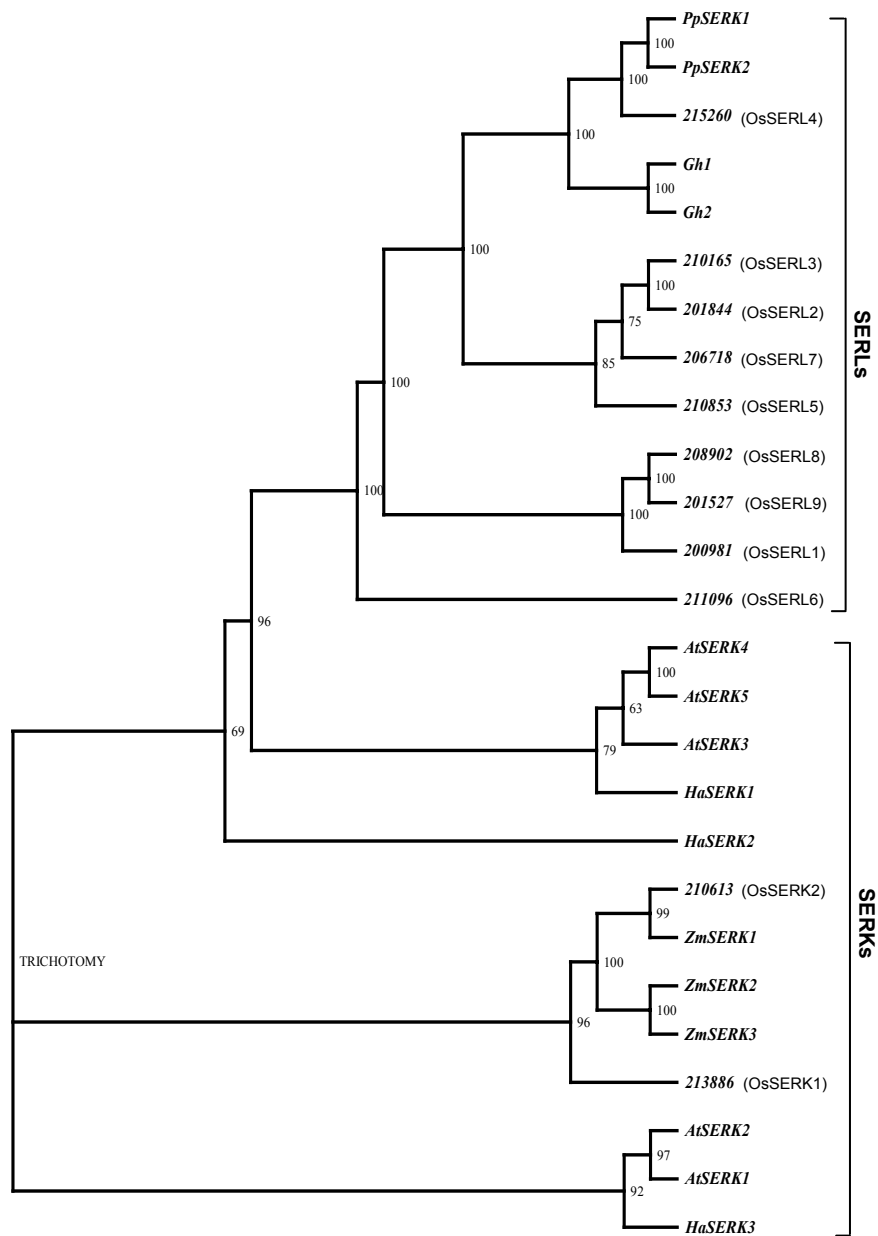

Supp. Fig. 2

Supplement: Supplementary file 2 [file 539402.f2.pdf]
