# Supplementary material for: Structural Characterization and Expression Analysis of the SERK/SERL Gene Family in Rice (Oryza sativa)
Source: Int J Plant Genomics. 2009 Sep 13;2009:539402. doi: 10.1155/2009/539402 (PMC2742738; doi:10.1155/2009/539402)
Supplement: Supplementary file 3 [file 539402.f3.doc]

**Supplementary Table 1.** Sequences of Real-Time PCR primers

| Name | Forward | Reverse |
| --- | --- | --- |
| OsSERL1 | CCA ACC GAA GGC ACT GTT TAA | CTG AAC ACC ACA GTG GTA AAA ACC |
| OsSERL2 | AAT GCC TCA CGA TCC AAT CTC T | CGC CAA CAC GAC CAG AAA T |
| OsSERL3 | GGC GTA AGC CGA ATC TGT ACT T | GTT CCA GGA CAA TTC TAT CTA CTC TAC AAA |
| OsSERL4 | TCA CCT TCG GTC GCT GCT A | TGC CTG GAC CAG CAA TGA C |
| OsSERL5 | ACT GCG TAC GTC CTG ATG CAT | ACT AAG AGC CAA CCA TAC ATA TAC ATA CAT G |
| OsSERL6 | GGA CTA TGA AGG GTG GTT CTT TTG | GTT TTT TTA AGA ATG GCA AGT GTT CTG |
| OsSERL7 | CTG CTC CCC GGA TTT CCT | TGC CGA TGC TTG GTG AGA |
| OsSERL8 | CCC TGA TCT CGA GCA AAC TTT T | CAG AAT TCA GAA GAA GCC CAT ACA |
| OsSERL9 | GTC CTC ACG GTT TGC ATA AGT AAC T | ACG AGA TGT AGC TGC ACG ACA TT |
| OsSERK1 | GTG CCG CCT TGG ATA TCG | GCA AGC TTC AGG ACA ATA GTT CCT |
| OsSERK2 | CTG GAG GGA AAA ATG GTT TAC TTG TA | GTG CAG AGC CTA CTT GAA AAT TAC C |
| *Actin* | CAG CCA CAC TGT CCC CAT CTA | AGC AAG GTC GAG ACG AAG GA |
